# Molecular detection of airborne *Emergomyces africanus*, a thermally dimorphic fungal pathogen, in Cape Town, South Africa

**DOI:** 10.1371/journal.pntd.0006174

**Published:** 2018-01-22

**Authors:** Ilan S. Schwartz, Josh D. McLoud, Dilys Berman, Alfred Botha, Barbra Lerm, Robert Colebunders, Estelle Levetin, Chris Kenyon

**Affiliations:** 1 Max Rady College of Medicine, University of Manitoba, Winnipeg, Manitoba, Canada; 2 Global Health Institute, University of Antwerp, Antwerp, Belgium; 3 Biological Sciences, University of Tulsa, Tulsa, Oklahoma, United States of America; 4 University of Cape Town, Cape Town, Western Cape, South Africa; 5 Department of Microbiology, Stellenbosch University, Stellenbosch, Western Cape, South Africa; 6 Department of Clinical Sciences, Institute of Tropical Medicine, Antwerp, Belgium; Universidad de Antioquia, COLOMBIA

## Abstract

*Emergomyces africanus* is a thermally dimorphic fungus that causes a systemic mycosis in immunocompromised persons in South Africa. Infection is presumed to follow inhalation of airborne propagules. We developed a quantitative PCR protocol able to detect as few as 5 *Es*. *africanus* propagules per day. Samples were collected in Cape Town, South Africa over 50 weeks by a Burkard spore trap with an alternate orifice. We detected *Es*. *africanus* in air samples from 34 days (10%) distributed over 11 weeks. These results suggest environmental exposure to airborne *Es*. *africanus* propagules occurs more commonly in endemic areas than previously appreciated.

## Author summary

*Emergomyces africanus* is a recently described dimorphic fungus that causes a serious and often fatal disease in immunocompromised patients in South Africa. Infection is presumed to occur via inhalation of infectious propagules; however, no attempts have yet been made to detect this fungus in the air. We developed a highly specific and sensitive protocol for the molecular detection and quantification of *Es*. *africanus*, and tested air samples collected over a 50-week period from an urban area of Cape Town, Western Cape, South Africa, where the disease is endemic. We detected *Es*. *africanus* in the air on 10% of all days sampled, suggesting that exposure to this fungus is common in this area. This study lays the groundwork for further investigations that might explore environmental, host, and pathogen factors that influence the development of this fungal disease.

## Introduction

*Emergomyces africanus* is an emerging opportunistic dimorphic fungal pathogen that causes emergomycosis, a systemic and often-fatal HIV-associated mycosis in South Africa [1,2]. It is a member of a newly-described genus within the family Ajellomycetaceae called *Emergomyces*, so-named because of the striking appearance or recognition of new dimorphic fungal pathogens reported globally [2]. In addition to *Emergomyces africanus*, which has been reported from South Africa and Lesotho [1–3], the genus includes *Emergomyces pasteurianus* (formerly *Emmonsia pasteuriana*, reported from Europe, Asia and Africa [4–10]) and *Emergomyces orientalis* (reported from China [11]). Similar but distinct fungi have also been reported from North America [12].

Although, the true incidence is unknown, cases of disease caused by *Emergomyces* species other than *Es*. *africanus* are uncommon, with only 13 cases reported to date. On the other hand, disease caused by *Es*. *africanus* is relatively common: although only described in 2013 (as *Emmonsia* species [1]), *Es*. *africanus* is now recognized to cause the most frequently diagnosed dimorphic fungal infection in South Africa [10]. In Cape Town, a clinical and laboratory surveillance study at public hospitals over a 15-month period identified 14 cases of culture-proven emergomycosis [13].

Emergomycosis is an opportunistic infection of immunocompromised hosts. In South Africa, one patient was a kidney transplant recipient, and the remainder have occurred in patients with advanced HIV infection (among whom the median CD4 lymphocyte count was 16 cells/μL) [1,3,14,15]. Patients most commonly present with widespread skin lesions and pulmonary disease [3], and the disease often only becomes clinically apparent after the initiation of antiretroviral therapy [3,13]. The reported case-fatality ratio is 50% [3].

Most patients with emergomycosis caused by *Es*. *africanus* have been diagnosed in the Western Cape province [3], although cases have been reported from 6 of 9 South African provinces and Lesotho [3,10].

The ecological niche of *Es*. *africanus* is still being elucidated, although an environmental reservoir of the mycelial phase in the soil is supported by molecular detection of *Es*. *africanus* in 30% of soils sampled from the Western Cape [16]. Nonetheless, most patients diagnosed with emergomycosis do not report occupational or other frequent exposure to soil [13]. Based on what is known of the pathogenesis of most other dimorphic fungal infections [17], *Es*. *africanus* infection likely follows inhalation of conidia or other infectious propagules. However, no attempts have been made to detect *Es*. *africanus* in the air.

We developed a protocol for the molecular detection and quantification of *Es*. *africanus* propagules from air samples, and used this to evaluate air samples collected from Cape Town, Western Cape.

## Methods

### Sampling

We sampled airborne propagules using a Hirst-type volumetric 7-day spore trap (Burkard Manufacturing Co. Ltd., Rickmansworth, Hertfordshire, England) [18] fitted with an alternate orifice (Burkard) [19]. This spore trap samples air continuously at a rate of 10 l/min (14.4 m^3^/day) [18]. The spore trap was set up on the roof of a building 4 m high in Bellville, an urban area of Cape Town, Western Cape, South Africa near where cases of *Es*. *africanus* infection have been diagnosed [3]. Permission was provided for sampling by the Air Pollution Department of the City of Cape Town, the owner of the property. Sampling took place over a period of 50 weeks from 15 September 2015 to 29 August 2016. Melinex tape was fixed to the sampler drum, greased with petroleum jelly and replaced weekly. Tapes were stored in individually labeled storage boxes at room temperature and shipped to the Aerobiology Lab at the University of Tulsa for molecular analysis. For processing, each Burkard tape was cut into 7 48-mm segments, with each segment representing a 24-hour period. The 48-mm segments were temporarily fixed on microscope slides labeled with the sampling date.

### DNA extraction from air samples

Each 48-mm tape segment containing the daily air sample was removed from the slide, cut into small strips and loaded into a sterile 2 ml bead-beating tube containing 0.3–0.4 g of 0.5 mm glass beads. The DNA extraction protocol was a variation of the cetrimonium bromide (CTAB) method [20]. A 2% CTAB extraction solution (pH 8.0) was added to the tube at a volume of 500 μl, along with 10 μl of β-mercaptoethanol. The tubes were loaded into the bead beater and shaken for 3 min at max speed. Tubes were incubated at 70°C for 1 hr before being centrifuged at 15,000 *g* for 1 min. The supernatant was transferred to a 2-ml centrifuge tube and centrifuged at 15,000 *g* for 10 min. The supernatant was transferred to another tube and 500 μl of chloroform:isoamyl-alcohol (24:1) was added, and vortexed for 10 sec. To separate the phases, tubes were centrifuged at 15,000 *g* for 10 min. The upper aqueous phase was collected and transferred to a clean tube with 100 μl of 5 M sodium acetate and 500 μl of ice-cold isopropyl, and the tubes vortexed for 5 sec. Tubes were stored at -20°C overnight. The next day, tubes were centrifuged at 15,000 *g* for 15 min at 4°C to pellet DNA. The supernatant was removed and discarded before the DNA pellet was washed with 800 μl ice-cold 70% ethanol at 4°C and centrifuged at 15,000 *g* for 10 min at 4°C. The supernatant was removed and the DNA pellet was air dried for ~30 min. The DNA pellet was suspended in 50 μl of enzyme free water and vortexed for 5 s and centrifuged at 15,000 *g* for 10 min before the DNA extract (from the daily air sample) was stored at -20°C.

For each week, fractions of the DNA from 7 daily air samples were pooled to produce a weekly air sample, which allowed testing of daily and weekly samples. A 15 μl aliquot of each 50 μl daily air sample (30%) was transferred to a 2-ml centrifuge tube, which produced a weekly sample with a final volume of 105 μl. Both the daily and weekly air sample DNA extracts were stored at 4°C until analyzed.

### qPCR assay development

qPCR assays specific for *Es*. *africanus* isolates (CBS 136730 and CBS 136260) were developed for regions of the nuclear ribosomal internal transcribed spacer (ITS) and the β-tubulin gene. All accessions of *Es*. *africanus* and gene constructs of closely related species used in this study are listed in S1 and S2 Tables. Gene constructs were manufactured by Eurofins MWG Operon USA, Louisville, KY. DNA sequence alignments were performed using the Multiple Sequence Comparison by Log-Expectation (MUSCLE) algorithm implemented in European Bioinformatics (EMBL-EBI; http://www.ebi.ac.uk) web browser. An alignment of *Es*. *africanus* JX398299 (formerly *Emmonsia* sp.), *Es*. *pasteurianus* KR150770 (formerly *Ea*. *pasteuriana*), *Emmonsia crescens* AF038336, *Histoplasma capsulatum* NR_149341, *H*. *capsulatum* KX646004, *Ajellomyces capsulatus* KM361509, *A*. *capsulatus* AF322386, *Ajellomyces dermatitidis* HQ026734, *Paracoccidioides brasiliensis* AY374339, *P*. *brasiliensis* JQ675762, *Ajellomyces grisea* AY527404, *Blastomyces percursus* KY195964, *B*. *percursus* KY 195963, Onygenales sp. KX148665, and Onygenales sp. KX148661 was performed *in silico* to test the specificity of the primers and probe for the *Es*. *africanus* assay.

TaqMan (Applied Biosystems, Foster City, CA) primer and probe design was performed manually using the alignment files with the following criteria: the maximum melting temperature differences between primers used in an assay were ±1.6°C and probes >7.5°C when compared to primers. The resulting primers and probe are shown in Table 1; these were manufactured by Eurofins MWG Operon USA. The probe was labeled with a fluorescein dye (6-FAM) at the 5’ end and a Black Hole Quencher 1 (BHQ-1) nonfluorescent quencher.

**Table 1:** The primers and probe used in this study.

Name	Tm	Sequence 5’-3’
**EmeITS(F)**	**56.0**	CTGGCCACCCTTGTCTAT
**EmeITS(R)**	**57.6**	GACGTCAATCTTTAACCAGTGTTT
**EmeITSprobe**	**65.5**	[6-FAM]TCTTGGCTCTCCGGGCTCGC[BHQ-1]
**EmeB-tubulin(F)**	**66.4**	AGGGCCATTACACCGAAGGTGC
**EmeB-tubulin(R)**	**66.3**	AAGTGGCCATCATGCGATCTGGG
**SepRef1(F)**	**64.5**	GGACTTCCAAGGCAGGTACATG
**SepRef1(R)**	**64.5**	GGGAAGTCCATCCAGCGATAGTAA

### TaqMan species-specific assay validation / qPCR assay for test samples

The specificity of the *Es*. *africanus* qPCR assay was validated *in silico* using the BLAST alignment tool in NCBI. The *in silico* specificity was also validated against the NCBI nucleotide database and there were no closely related isolates homologous for the assay target, including two isolates of the dimorphic fungus *Blastomyces percursus* (CBS 139878 and NCPF 4091) that is endemic to South Africa [2]. The specificity of the primers EmeITS(F) and EmeITS(R) was further validated *in vitro* by end-point PCR using *Es*. *africanus* gDNA and gene constructs from other closely related species (S1 Table), as well as a mock community of fungal species potentially present in air samples. The mock community consisted of gDNA from *Alternaria* sp., *Aspergillus niger*, *Cladosporium* sp., *Epicoccum nigrum*, and *Trichoderma* sp. Each member of the mock community was isolated from air samples and identified by morphology. A single fragment of 129 bp was amplified from the *Es*. *africanus* gDNA. No product was observed with the gene constructs of *Es*. *pasteurianus* or *Ea*. *crescens* (accessions KR150770 and AF038336, respectively) or the air sample mock community gDNA. Development of the qPCR assay included the addition of the TaqMan probe.

Quantitative PCR using the *Es*. *africanus* ITS primers described above and Taqman probe was performed with a StepOnePlus System (Applied Biosystems, Foster City, CA). All reactions were performed in a final volume of 25 μl and contained 12.5 μl of 2× TaqMan Gene Expression Master Mix (Applied Biosystems), 2.5 μl of each 5 μM primer solution, 2.5 μl of 2.5 μM TaqMan probe solution, and 5 μl of template DNA. PCR thermocycling conditions were set at 95°C for 15 min, 40 cycles at 95°C for 15 s and 61.9°C for 30 s. All air samples were tested in triplicate.

### TaqMan qPCR standard curve

To produce amplicon standards for the standard curve, end-point PCR was performed with the ITS primers using 20 ng of *Es*. *africanus* (CBS 136260) gDNA. Gel electrophoresis was used to remove the unincorporated nucleotides from the PCR amplicons; this was followed by column purification with Illustra GFX PCR DNA and Gel Band Purification Kit, isolating the amplicons. Purified amplicons were quantified with a Qubit 2.0 and dsDNA HS assay Kit. The quantified amplicon solution was diluted based on the mass of amplicon and concentration of amplicon in the initial solution (Applied Biosystems, 2003). This resulted in solutions containing 60,000, 6,000, 600, 60, and 6 target gene copies in 5 μl of solution, which were used to create the standard curve. The positive control was 20 ng/μl of gDNA and the negative control was water in replacement of the gDNA. For the qPCR assay, each 96-well plate included reactions for a standard curve, positive control, and negative control with three replicates for all reactions. In addition, for days positive for *Es*. *africanus*, quantification was repeated on a different day to increase the number of replicates and to confirm there were no false positives.

### Calculation of target copy number in gDNA

Deducing the number of *Es*. *africanus* cells or propagules from the number of target sequences detected by absolute qPCR required the knowledge of target copy number per cell. The β-tubulin gene is a single-copy gene [21–24]; therefore, the comparisons between the β-tubulin target and ITS targets for the same gDNA concentration enabled calculation of the number of ITS targets per genome. The ITS targets for our assay were normalized to the single β-tubulin target, allowing use of the amplicon standard curve to create an assay with a sensitivity below one propagule per 5 μl sample volume analyzed.

Standards were developed for an absolute qPCR β-tubulin SYBR Green assay. To produce known standards, end-point PCR was carried out with the β-tubulin and ITS primers using 20 ng of *Es*. *africanus* gDNA; each PCR product was column purified with Illustra GFX PCR DNA and Gel Band Purification Kit (GE Healthcare, Chicago, IL). Cleaned PCR product was quantified with a Qubit 2.0 and dsDNA HS assay Kit (Thermofisher Scientific, Waltham, MA), and each quantified PCR product was diluted based on mass of amplicon and concentration of amplicon in the initial solution [25]. This resulted in solutions containing 60,000, 6,000, 600, 60, and 6 target gene copies in 5 μl of solution for each gene. These solutions were used to generate qPCR reactions with final volumes of 20 μl with 10 μl of 2× PowerUp SYBR Green PCR Master Mix (Applied Biosystems, Foster City, CA), 2 μl of each 5 μM primer solution, 1 μl of enzyme free water, and 5 μl of standard template solution. Quantitative PCR was performed with a StepOnePlus System (Applied Biosystems). PCR thermocycling conditions were set at 95°C for 2 min, 40 cycles at 95°C for 15 s and 60°C for 1 min. Fluorescence was read at the end of each extension step and there were four replicates for all standards.

**Fig 1:**
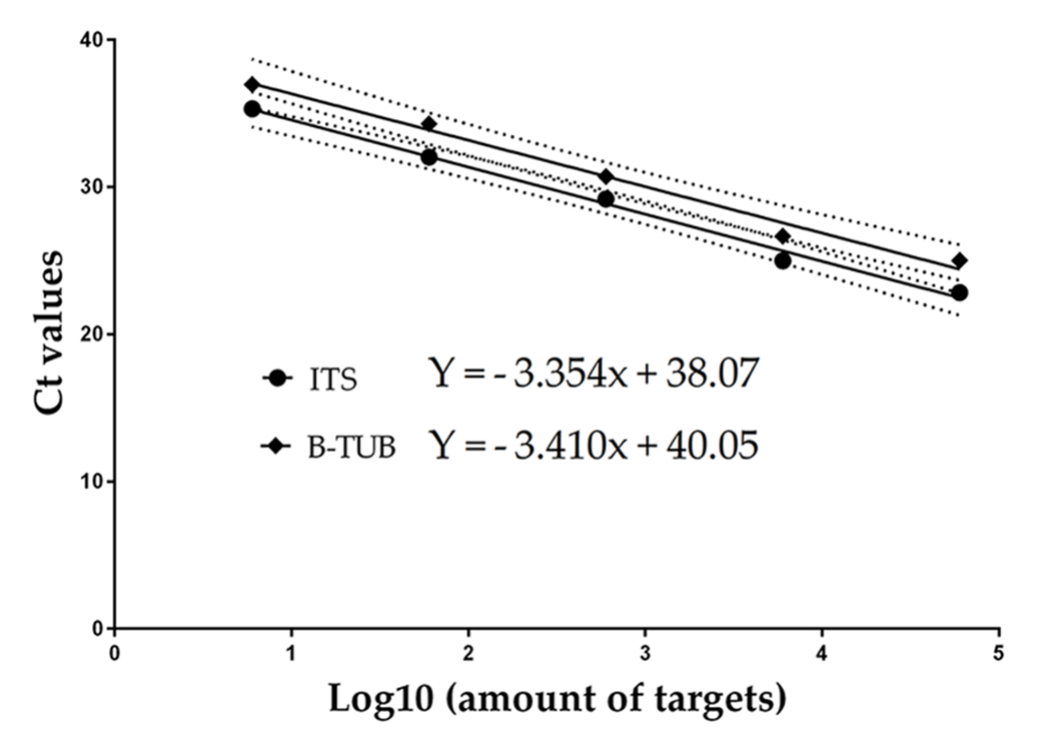
Standard curves from the amplification of 10-fold dilutions of ITS and β-tubulin amplicons. These curves showed a linear relationship across the whole range (ITS, R^**2**^ = 0.99 and β-TUB, R^2^ = 0.98), between the log_10_ value of the amplicon concentrations and the threshold cycles. The high coefficient of determination indicated low intra-assay variability. The dotted lines are the 95% CI of the coefficient of determination, which show that there is no significant difference between the 2 standard curves used to generate the mean target copy number per genome.

An absolute qPCR β-tubulin SYBR Green assay was used to test the mean copy number of targets from two isolates (CBS 136260 and CBS 136730) of *Es*. *africanus* gDNA. Genomic DNA reactions containing 60, 6, and 0.6 ng were performed in a final volume of 20 μl, containing 10 μl of 2× PowerUp SYBR Green PCR Master Mix, 2 μl of each 5 μM primer solution (Table 1), 3 μl of enzyme free water, and 3 μl of template DNA. There were four replicates for each gDNA concentration. For the 0.6 ng reaction only isolate CBS 136260 was used. The quantity of ITS and β-tubulin target copy numbers were determined by comparing the Ct values of different gDNA concentrations to those of the known target number of the standard curve (Fig 1).

The absolute quantification resulted in ITS target copy numbers of 5.97x10^8^ and 5.04x10^8^ (for the 60 ng reactions) 1.33x10^6^ and 1.03x10^5^ (6 ng reactions) and 2.98x10^3^ (0.6 ng reactions) and β-tubulin target copy number of 5.92x10^7^ and 5.09x10^7^ (60 ng), 9.08x10^4^ and 5.85x10^3^ (6 ng), and 1.08x10^2^ (0.6 ng) respectively. When ITS target numbers were divided by the β-tubulin target numbers for the same DNA concentration, the calculated ITS targets per genome from highest to lowest DNA concentration were 10, 10, 15, 18, and 28, respectively. The mean (n = 5) ± SE ITS target number using all gDNA concentrations was 16 ± 3 with a SD of 7.3, and after removal of two outliers was 12 ± 2 with a SD of 2.7. Accordingly, 12 ITS targets were used to determine the number of *Es*. *africanus* propagules. The SYBR Green quantification was followed with a melting curve analysis, which produced single peaks for both the ITS and β-tubulin amplicons (S1 Fig).

### PCR inhibition analysis

**Fig 2:**
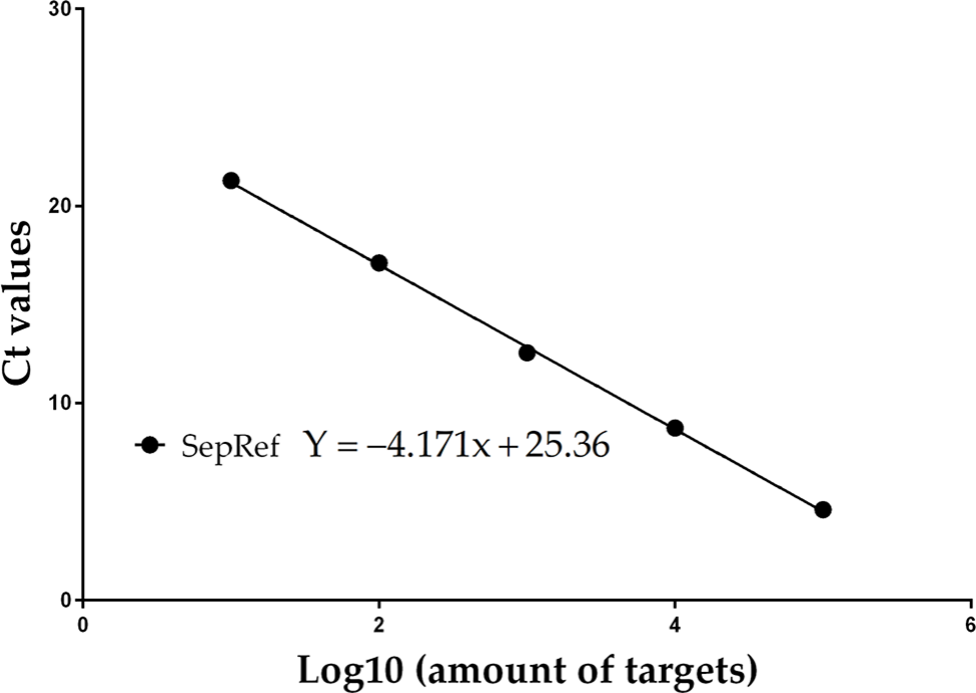
Standard curve for the SepRef (cuttlefish) inhibition assay (R^2^ = 0.98).

DNA extracts were tested for PCR inhibitors to confirm the ability to amplify DNA. The qPCR master mix was spiked with a known concentration of a gene construct (S1 Table) of the reflectin gene of cuttlefish (*Sepia officinalis*), an ocean living mollusk (the DNA of which should not be found in air samples), and the Ct value of the DNA extract from air samples tested [26]. All weekly air samples were amplified with cuttlefish primers (Table 1) and had a mean (± SD) Ct value of 11 ± 0.47 (Fig 2). When the daily air samples from the *Emergomyces* positive weeks were tested, 6 samples showed the presence of inhibitors. Samples were cleaned by repeating the chloroform: isoamyl alcohol and subsequent steps of the DNA extraction. After cleaning, all 77 daily samples amplified with cuttlefish primers and had a mean (± SD) Ct value of 10 ± 0.78.

### Analysis

**Fig 3:**
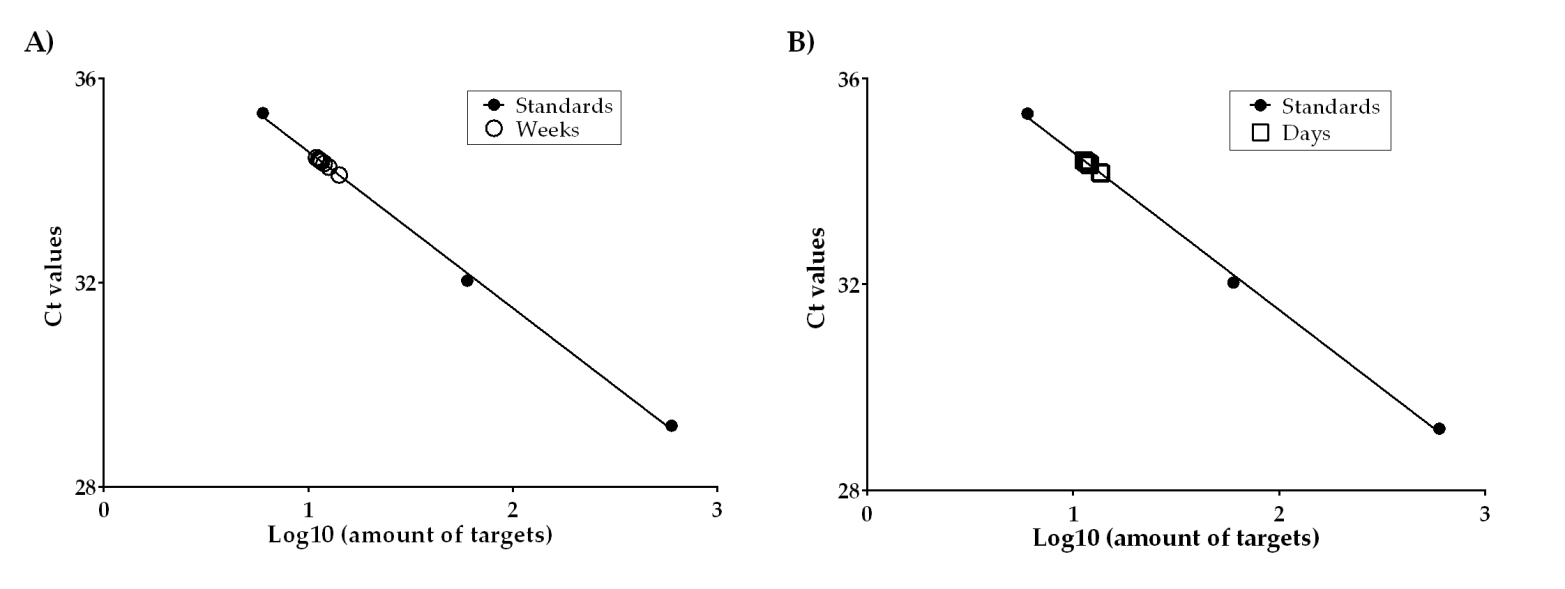
Mean ITS target number of quantifiable weekly and daily air samples for *Es*. *africanus* fit on the standard curve. **Note:** Five standards were used to generate the standard curve; however, only three standards are shown in these figures to increase the visibility and separation of the test samples. A) Weekly air samples B) Daily air samples.

StepOne Software v2.0 (Applied Biosystems) was used to determine the quantity of ITS target copy numbers for weekly (pooled daily samples) and daily air samples. The targets in the total DNA extract sample volume (105 μl for the weekly samples and 50 μl for the daily samples) and the resulting total propagules were determined by comparing the Ct values of gDNA concentrations from air samples to the Ct values of the standard curve (Fig 3A and 3B). The mean ITS target copy numbers were computed using all replicates of daily air samples (Fig 3B).

### Meteorological data

Daily meteorological data was obtained for the sampling period from South African Weather Service for Cape Town International Airport 6.8 km away. This included wind speed (collected at the times 0800, 1400 and 2000), daily maximum and minimum temperatures, and daily rainfall. Mean values for each week were plotted using GraphPad Prism version 6.00 against the number of days in that week that *Es*. *africanus* propagules were detected.

## Results

A standard curve for the qPCR assay was obtained from 5 different amplicon concentrations from 6 to 60,000 ITS target copies (Fig 1). There was amplification of all amplicon dilutions and the positive control. The limit of quantification was 6 ITS target copies. Therefore, target quantities with fewer than 6 were only used for qualitative assessments of the presence of *Es*. *africanus* DNA. No amplification was detected for the no-template control.

*Es*. *africanus* DNA was detected among weekly samples during 11 of 50 weeks. Ten of the 11 weeks produced ITS target numbers that were within the range of the standard curve (Fig 3A) and could be quantified (Table 2). During the 11^th^ week in which *Es*. *africanus* DNA was detected, fewer than 6 targets were amplified.

**Table 2:** Detection of *Es*. *africanus* propagules in weekly air samples.

Week	Mean copies of target gene detected in 5 μl aliquot amplified	Total propagules in pooled weekly sample (105 μl total volume)
6 Oct to 12 Oct 15	8	14
3 Nov to 9 Nov 15	10	17
24 Nov to 30 Nov 15	21	37
8 Dec to 14 Dec 15	3*	BLQ
24 May to 30 May 16	8	14
7 Jun to 14 Jun 16	7	12
14 Jun to 20 Jun 16	6	11
12 Jul to 18 Jul 16	7	12
19 Jul to 25 Jul 16	14	25
26 Jul to 1 Aug 16	36	63
9 Aug to 15 Aug 16	6	11

Below the limit of quantification (BLQ)

*This value was outside of the standard curve and unreliable for quantification

Aliquots of DNA from the daily air samples (n = 77) from the 11 positive weekly samples were analyzed for *Es*. *africanus*. Initially, there were 31 daily samples positive for *Es*. *africanus* DNA. Although the weekly samples did not show PCR inhibition, all daily air samples from the *Es*. *africanus* positive weeks were also tested for the presence of PCR inhibition. DNA extracts from 6 daily air samples were determined to contain PCR inhibitors; after cleaning, the 6 samples were retested for *Emergomyces*. Three of the 6 previously inhibited daily samples were positive for *Es*. *africanus*, bringing the total number of *Es*. *africanus* positive daily samples to 34; however, only 11 daily samples were within the range of the standard curve and quantified (Table 3). This left a total of 23 daily samples that were positive for *Es*. *africanus* DNA but outside the standard curve (Fig 3B) and thus below the limit of quantification (S2 Fig).

**Table 3:** Detection of *Es*. *africanus* propagules in daily air samples.

Date	Mean copies of target gene detected in 5 μl aliquot amplified	Total propagules in air sample
7 Oct 15	15	13
13 Jun 16	14	12
16 Jun 16	6	5
13 Jul 16	8	7
19 Jul 16	13	11
20 Jul 16	6	5
21 Jul 16	10	8
24 Jul 16	6	5
31 Jul 16	31	26
1 Aug 16	29	24
14 Aug 16	7	6

**Fig 4:**
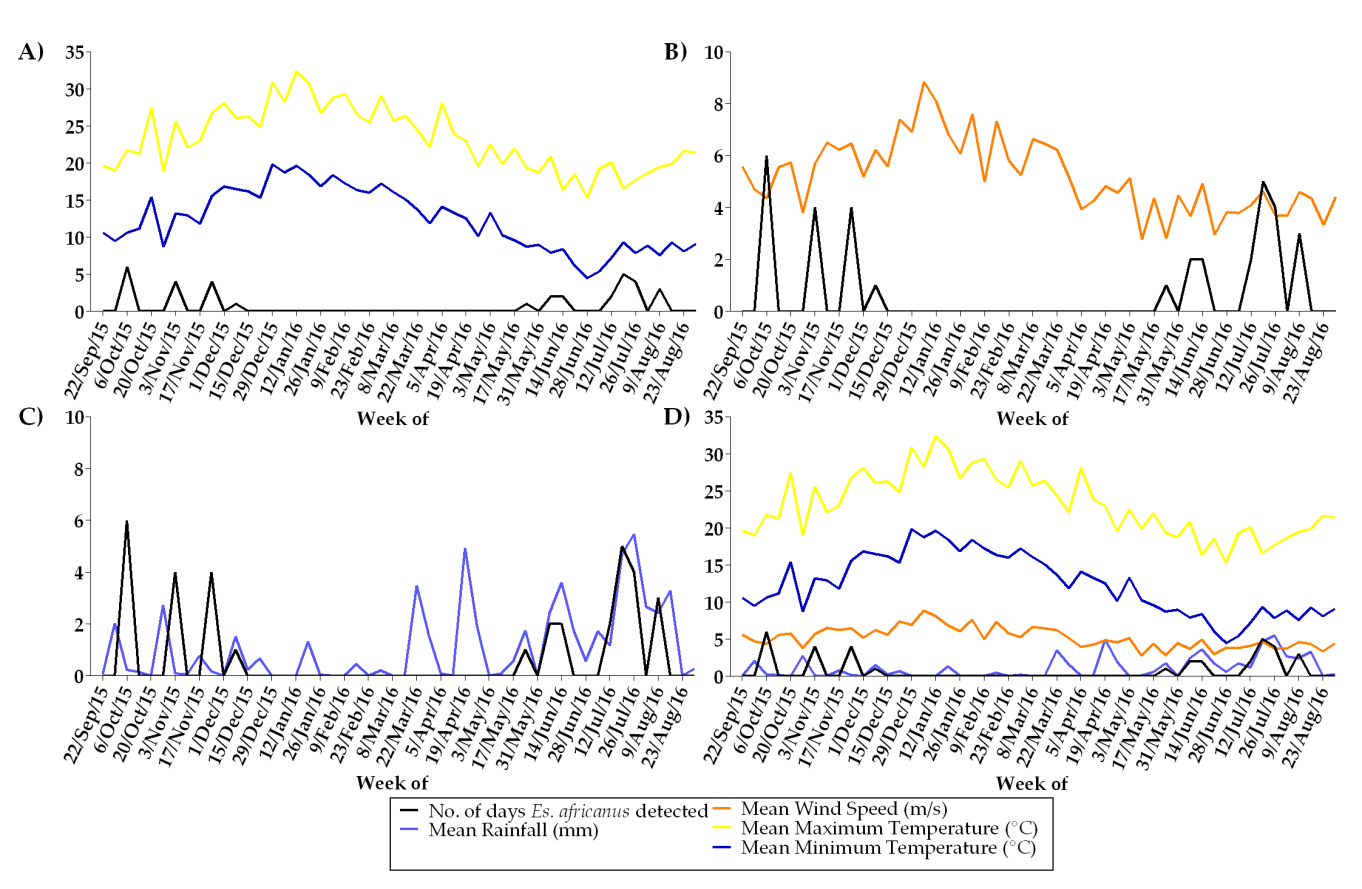
Weather conditions in relation to the detection of airborne propagules of *Es*. *africanus*. The number of days per week in which *Es*. *africanus* was detected using qPCR is plotted against (A) weekly mean maximum and minimum temperature, (B) wind speed (C) rainfall, and (D) all variables.

The respective relationships between the detection of *Es*. *africanus* (34 days during the 11 positive weeks) at our sampling site and the prevailing mean wind speed, maximum and minimum daily temperatures, and mean rainfall at Cape Town International Airport are demonstrated in Fig 4. Although not enough *Es*. *africanus* positive weeks were detected to attempt statistical correlations with meteorological variables, it is possible that cooler temperatures (during winter and spring) and rainfall coincided with *Es*. *africanus* propagules in the air samples.

## Discussion

*Emergomyces africanus* is an important cause of an AIDS-related mycosis in South Africa [13]. We developed a qPCR assay that is highly specific and sensitive for the detection and quantification of *Es*. *africanus* in spore trap air samples, and demonstrated the frequent airborne circulation of *Es*. *africanus* in an industrial area of Cape Town.

Other studies have used different protocols for molecular detection of *Es*. *africanus* in South Africa. Using a conventional PCR, Cronjé *et al* failed to detect *Es*. *africanus* in tissues of 1402 small terrestrial mammals from across South Africa [27]. Schwartz *et al* used a nested (conventional) PCR strategy and found *Es*. *africanus* in 30% of soil samples assessed [16]. The main advantage of the protocol presented here is the ability of qPCR to quantify the number of propagules present. Additional advantages of our assay include the high specificity for *Es*. *africanus*, which could be clearly distinguished from other closely related fungi in addition to a distantly related mock community. Moreover, our assay was highly sensitive, detecting as few as five propagules per day; the minimum inhaled dose of *Es*. *africanus* propagules required to cause infection is unknown.

Limitations of our study include the fact that only a single spore trap was used from a single location. Additionally, the climatic data in our study is from a meteorological station 6.8 km away from the sampling site, and conditions at the spore trap may have been different from those measured. A limitation of the Burkard spore trap is the inability to use culture-based analyses [28]. Consequently, we cannot definitively conclude the infectivity of the detected propagules. Alternatively, the Burkard spore trap can be a robust sampling technique that allows molecular analyses of samples [29,30]. Our study is useful in demonstrating that airborne propagules of *Es*. *africanus* can be detected. Future investigations should include multiple concurrent spore traps in different locations to further characterize the range of detection as well as clarify the factors associated with the presence of airborne *Es*. *africanus* propagules.

That *Es*. *africanus* propagules were frequently detected in an urban setting suggests that exposure to *Es*. *africanus* is common in Cape Town (and perhaps other areas where infection has been diagnosed). While emergomycosis has only been reported in patients who are immunocompromised, many similarly immunocompromised patients do not develop the disease [13]. Further research should consider the question of which environmental, host and/or pathogen factors influence whether infection leads to disease.

## Supporting information

S1 Table Accessions of partial ITS1; 5.8S rRNA gene; partial ITS2 in NCBI database used to test specificity of *Emergomyces africanus* assay *in silico* and *in vitro* and the accession used for the inhibitor testing. (PDF)

S2 Table Accessions of partial β-tubulin gene used to develop a β-tubulin assay for *Emergomyces africanus* to determine target copy number. (DOCX)

S1 Fig Melting curve analysis confirming specificity of PCR reactions. A) ITS amplicon melting curve. B) β-tubulin amplicon melting curve.
(TIF)

S2 Fig Number of positive days in each of the 11 weeks during which *Emergomyces africanus* was detected by PCR but at numbers below the limits of quantification. (TIF)
