# Correction: Molecular detection of airborne *Emergomyces africanus*, a thermally dimorphic fungal pathogen, in Cape Town, South Africa

**DOI:** 10.1371/journal.pntd.0006468

**Published:** 2018-05-02

**Authors:** The PLOS Neglected Tropical Diseases Staff

An unrelated file was inadvertently published as S1 Table. Please view the correct S1 Table below. The publisher apologizes for the error.

## Supporting information

S1 Table Accessions of partial ITS1; 5.8S rRNA gene; partial ITS2 in NCBI database used to test specificity of *Emergomyces africanus* assay *in silico* and *in vitro* and the accession used for the inhibitor testing. (DOCX)
