# Supplementary material for: Molecular detection of airborne Emergomyces africanus, a thermally dimorphic fungal pathogen, in Cape Town, South Africa
Source: PLoS Negl Trop Dis. 2018 Jan 22;12(1):e0006174. doi: 10.1371/journal.pntd.0006174 (PMC5800596; doi:10.1371/journal.pntd.0006174)
Supplement: S2 Table — (DOCX) [file pntd.0006174.s002.docx]

**S2 Table. Accessions of partial β-tubulin gene used to develop a β-tubulin assay for *Emergomyces africanus* to determine target copy number.**

| Species | Accession number | Type | Sequence* |
| --- | --- | --- | --- |
| *Emmonsia parva* | HF563667 | sequence | CGGTCCCTTTGGCCAGCTCTTCCGCCCTGACAACTTCGTTTTCGGCCAGTCTGGTGCTGGAAACAACTGG  GCCA**AGGGCCATTA**T**AC**T**GA**G**GG**C**GC**TGAGCTTGTCGACCAGGTCATCGATGTCGTTCGCCGTGAGGCTGAAGGCTGCGACTGCCTCCAGGGTTTCCAGATCACTCACTCCCTTGGTGGTGGTACCGGTGCCGGTATGGGTACCTTGTTGATCTCCAAGATCCGTGAGGAGTT**CCCAGA**C**CG**T**ATGATGGCCACTT**TCTCCGTCGTTCCTTCGCCAAAGGTTTCCGACACCGTCGTCGAGCCTTACAATGCCACCCTCTCCATCCATCAGCTCGTGGAGC |
| *Emergomyces africanus [Emmonsia sp.* AB-2012-1] | HF563663 | sequence | CGGTCCCTTTGGCCAGCTCTTCCGTCCAGACAACTTCGTTTTCGGGCAGTCTGGTGCTGGAAACAACTGG  GCCA**AGGGCCATTACACCGAAGGTGC**TGAGCTGGTTGACCAGGTCATCGATGTCGTGCGCCGTGAAGCTGAAGGCTGCGACTGCCTCCAGGGTTTCCAGATCACTCACTCCCTTGGTGGTGGTACCGGTGCTGGTATGGGTACCTTGTTGATTTCCAAGATCCGTGAGGAATT**CCCAGATCGCATGATGGCCACTT**TCTCTGTCGTGCCTTCGCCAAAGGTTTCTGACACCGTTGTCGAGCCTTACAATGCCACCCTCTCAATCCATCAACTCGTTGAGC |

*Bold nucleotides are homologous with primers and probes while underlined nucleotides are non-homologous
