# Supplementary figures and images for: Molecular detection of airborne Emergomyces africanus, a thermally dimorphic fungal pathogen, in Cape Town, South Africa
Source: PLoS Negl Trop Dis. 2018 Jan 22;12(1):e0006174. doi: 10.1371/journal.pntd.0006174 (PMC5800596; doi:10.1371/journal.pntd.0006174)

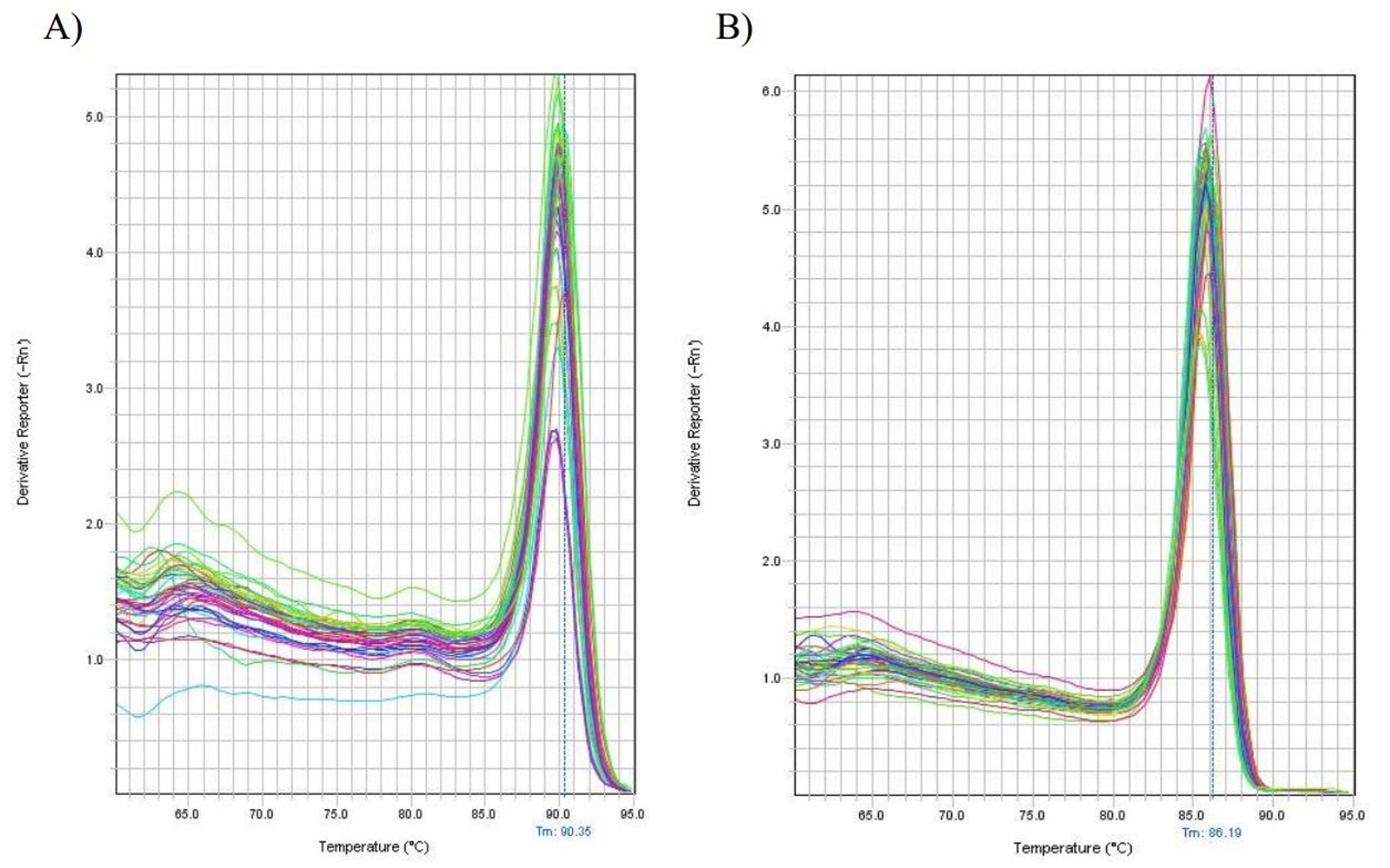

Supplement: S1 Fig — A) ITS amplicon melting curve. B) β-tubulin amplicon melting curve. (TIF) [file pntd.0006174.s003.tif]

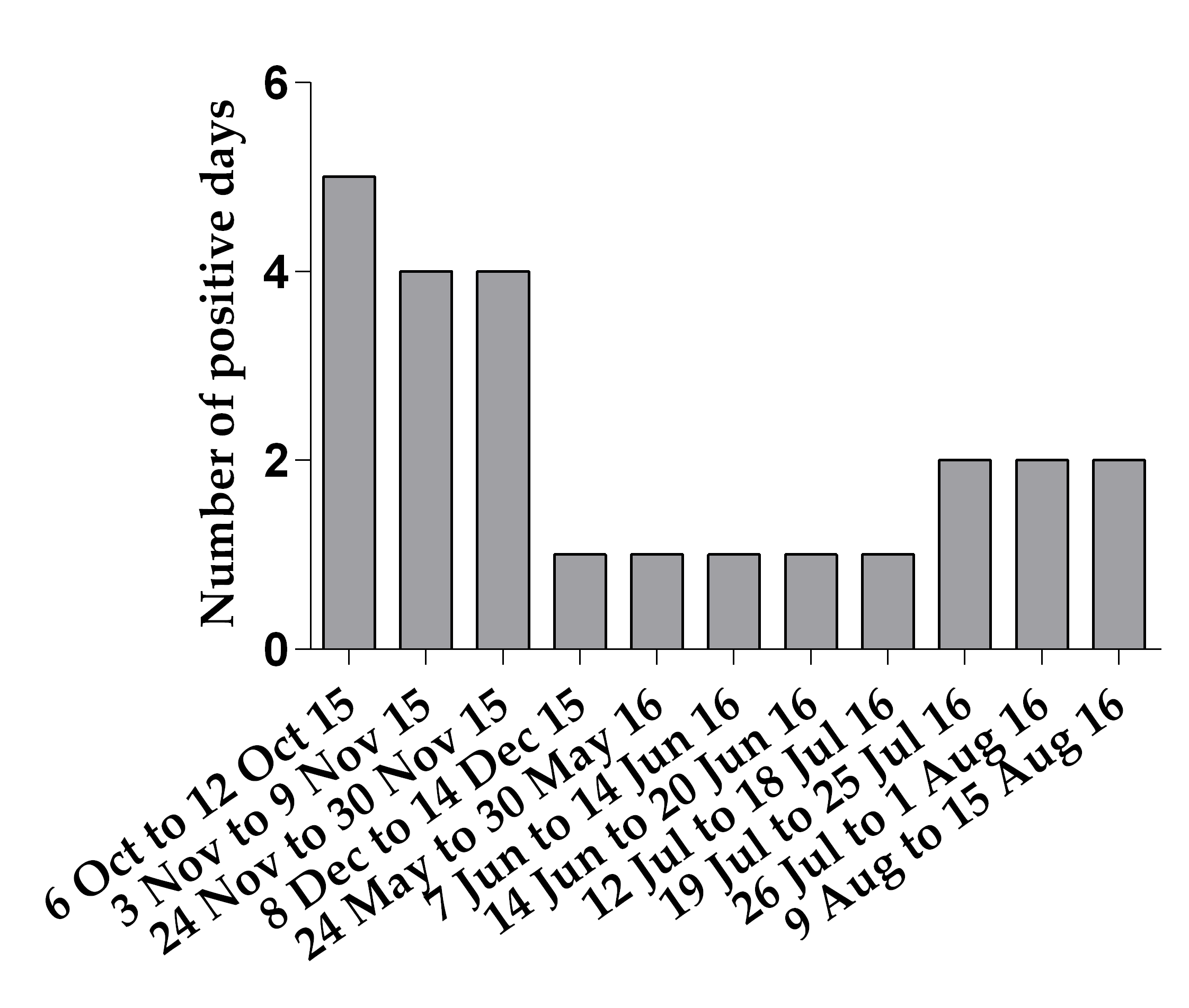

Supplement: S2 Fig — (TIF) [file pntd.0006174.s004.tif]
