# Supplementary material for: Correction: Molecular detection of airborne Emergomyces africanus, a thermally dimorphic fungal pathogen, in Cape Town, South Africa
Source: PLoS Negl Trop Dis. 2018 May 2;12(5):e0006468. doi: 10.1371/journal.pntd.0006468 (PMC5931619; doi:10.1371/journal.pntd.0006468)
Supplement: S1 Table — (DOCX) [file pntd.0006468.s001.docx]

**S1 Table. Accessions of partial ITS1; 5.8S rRNA gene; partial ITS2 in NCBI database used to test specificity of *Emergomyces africanus* assay *in silico* and *in vitro* and the accession used for the inhibitor testing.**

| Species | Accession number | Type | Sequence* |
| --- | --- | --- | --- |
| *Emergomyces africanus*  [NCBI as *Emmonsia* sp. AB-2012-1] | JX398299 | gDNA | GGTCGGTCTCCACCGGGGACCCGGCCCCTCCAC**CTGGCCACCCTTGTCTAT**CTCACCTGTTGCTTCG  GCGGGCCTGCAGCGATGCTGCCGGGGGAGC**TCTTGGCTCTCCGGGCTCGC**GCCCGCCGGGGACAC  CGTAGA**AAACACTGGTTAAAGATTGACGTC**TGAGACTATAACTGTAATAAGTTAAAACTTTCAACA  ACGGATCTCTTGGTTCCGACATCGATGAAGAACGCAGCGAAATGCGATAAGTAATGTGAATTGCAG  AA |
| *Emergomyces pasteuriana*  [NCBI as *Emmonsia pasteuriana*] | KR150770 | Gene construct | CGGGACCCCGGCCCCTCCAC**CTGGCCACCCTTGTCTA**CCTCACCTGTTGCTTCGGCGGGCCTGCAG  CGA**T**GC**TG**C**C**GGGGGA**G**CT**TCGC**CTCCCCGGGCCCGTGCCCGCCGGGGACACCGTAGA**AAAC**T**CT**  **GGTTAAAGATTGACGTC**TGAGAATATA |
| *Emmonsia crescens* | AF038336 | Gene construct | CCCTCAC**CTGGCCACCCTTGTCTAT**TCTACCTGTTGCTTCGGCGGGCCTGCAGCAATGCTGCCGGGG  GAG**TCTT**CT**CTC**C**CCGGGC**C**CGC**GCCCGCCGAGGACACCCTAG**AA**C**C**T**C**C**GGTTAAAGATTGACGT**  **C**TGAGACATAACTATAATCAGTTAAA |
| *Sepia officinalis* | HE866979 | Gene construct | GAATGACCAT**GGACTTCCAAGGCAGGTACATG**TAGAAGGACGCTACATGGACAGGACTCCCAGGG  CAGAATGGTCGACCCCAGATACTACTCCATGTTCAACTACGGCTGAGATGATGGATGAGTGACAGG  TACAACAA**TTACTATCGCTGGATGGACTTCCC**TCGCGATATGTCCAACTGGCAGAAGAGGTACAT |

*Bold nucleotides are homologous with primers and probes while underlined nucleotides are non-homologous
